# Purely extradural meningioma presenting as an incidental sacro-iliac joint mass

**DOI:** 10.1016/j.radcr.2026.07.017

**Published:** 2026-08-07

**Authors:** Aurélien Dulaurent, Alexandre Salem, Kerman Zyani, Louis-Romée Le Nail, Loïc Bouilleau

**Affiliations:** a Radiology Department, CHU de Tours, Chambray-les-Tours, France; b Anatomical Pathology Department, CHU de Tours, Chambray-les-Tours, France; c Orthopedic Surgery Department, CHU de Tours, Chambray-les-Tours, France

**Keywords:** Meningioma, Sacroiliac, Sacral, Extradural, MRI, CT

## Abstract

We report the case of an asymptomatic 32-year-old woman in whom a 38-mm right sacroiliac mass was incidentally discovered on computed tomography. Computed tomography demonstrated a well-defined enhancing lesion centered on the right sacral ala and sacroiliac joint with sclerotic margins, without cortical destruction or presacral extension. Magnetic resonance imaging showed a relatively homogeneous mass, iso- to hypointense on T1-weighted images, hyperintense on short tau inversion recovery sequences, and demonstrating intense homogeneous enhancement after gadolinium administration. Computed tomography-guided biopsy revealed a spindle-cell proliferation with diffuse somatostatin receptor 2A (SSTR2A), epithelial membrane antigen, and progesterone receptor expression, and a low Ki-67 index (1%), consistent with a World Health Organization grade 1 meningioma. One-year imaging follow-up demonstrated strict stability, and conservative management was maintained.

## Introduction

Meningiomas are typically benign tumors arising from arachnoid cap cells and account for approximately one-third of primary intracranial tumors [1]. Spinal locations represent a well-defined subgroup, most commonly occurring in the thoracic intradural compartment. In contrast, sacral involvement remains rare, and purely extradural forms are exceptional [2].

These atypical locations pose a significant diagnostic challenge. Clinical presentation is often nonspecific, and imaging features may overlap with those of more common lesions, particularly metastatic disease [3,4].

Extradural sacral meningiomas therefore constitute an extremely rare entity, with only a few isolated cases reported in the literature [[5], [6], [7]]. When present, clinical manifestations may include low back pain, pelvic pain, or radicular symptoms. In some cases, the lesion is discovered incidentally during imaging performed for an unrelated indication [8]. Radiologically, the appearance may mimic schwannoma, metastatic disease, primary bone tumors, solitary fibrous tumor, or other sacral masses. Consequently, preoperative diagnosis is rarely established with certainty, and imaging plays a pivotal role in narrowing the differential diagnosis and guiding therapeutic management.

On magnetic resonance imaging (MRI), these lesions typically present as well-circumscribed masses, iso- to hypointense on T1-weighted images, with variable signal intensity on T2-weighted sequences, and showing marked enhancement after gadolinium administration. However, the absence of an associated intradural component, as well as the possible presence of osseous remodeling or sacroiliac extension, may complicate interpretation. Computed tomography further refines the assessment by characterizing osseous architectural changes, such as peripheral sclerosis or cortical erosion. Definitive diagnosis relies on histopathological and immunohistochemical analysis. Owing to the rarity of these lesions, data regarding their natural history, optimal management, and recurrence risk remain limited [9,10].

We report an exceptional case of an extradural sacroiliac meningioma, detailing its imaging features and emphasizing the importance of including this entity in the differential diagnosis of sacral and sacroiliac masses.

## Case report

A 32-year-old woman underwent computed tomography (CT) as part of the evaluation for recurrent pyelonephritis. She reported no low back pain, radicular symptoms, or sensory disturbances.

**Fig. 1:**
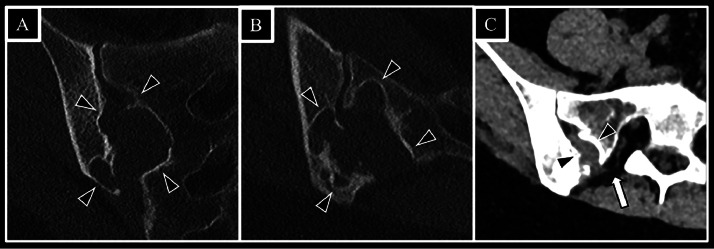
Incidental finding of a right sided sacro-iliac mass on CT. (A) Coronal oblique reconstruction, bone window; (B) axial oblique reconstruction, bone window; (C) axial reconstruction, soft tissue window; black arrowheads exhibit the meningioma, white arrow exhibit close contact with the dorsal ramus of S1.

CT imaging included a noncontrast abdominopelvic acquisition and a subsequent portal venous phase acquisition following intravenous administration of iodinated contrast agent. CT showed no evidence of pyelonephritis or urinary tract lithiasis. However, an incidental finding revealed an approximately 38 × 31 × 35 mm mass centered on the right sacroiliac joint and right sacral ala, demonstrating enhancement after intravenous contrast administration. The lesion exhibited sclerotic margins without cortical erosion, intralesional calcification, or presacral extension. The leading hypothesis was a slowly progressive lesion arising next to the posterior “ligamentous” compartment of the right sacroiliac joint, extending into the adjacent soft tissues with associated cortical scalloping of the neighboring osseous structures (Fig. 1). Multiplanar reconstructions showed close contact with the dorsal ramus of S1.

Following multidisciplinary discussion, MRI and CT-guided percutaneous biopsy were performed to further characterize this asymptomatic lesion.

MRI demonstrated a relatively homogeneous mass, iso to hypointense on T1-weighted images and hyperintense on short tau inversion recovery sequences, with intense and homogeneous enhancement after gadolinium administration. Sclerotic margins were noted, along with mild adjacent bone marrow edema, without foraminal extension.

Histological examination showed a spindle-cell tumor composed of bland fusiform cells arranged in parallel and intersecting fascicles within a fibrous stroma, admixed with vascular structures. The tumor cells displayed monotonous cytology, without significant nuclear atypia or necrosis. No chondroid or osteoid matrix was identified. Mitotic activity was low, with approximately 2 mitoses/mm².

Immunohistochemically, the tumor cells showed diffuse somatostatin receptor 2A (SSTR2A) expression, diffuse epithelial membrane antigen expression and diffuse nuclear progesterone receptor expression. The Ki-67 proliferation index was low, estimated at approximately 1%. S100 protein showed fairly diffuse staining, whereas SOX10 was negative in tumor cells. Cytokeratin AE1/AE3 and neurofilament were negative. Desmin and caldesmon were negative, and smooth muscle actin stained only exceptional cells and vascular structures. CD34 and ERG were negative in the spindle tumor cells and highlighted vascular structures only (Table 1).

**Table 1:** Immunohistochemical study performed on deparaffinized sections (automated Benchmark ULTRA, Ventana).

Antibody	Result(s)
S100 protein *(polyclonal, DAKO)*	Rather diffuse staining of tumor cells
SOX 10 *(clone SP267, CELLULES MARQUE)*	No staining of tumor cells
SSTR2A *(clone NB-49-015, Clinisciences)*	Diffuse staining of cells
Progesterone receptors *(clone 16, LEICA BIOSYSTEMS/NOVOCASTRA)*	Diffuse nuclear staining of cells
Neurofilament *(clone 2F11, DAKO)*	No staining
Ki-67 *(clone 30-9, VENTANA)*	Weak, estimated 1%
Cytokératine AE1/AE3 *(clone AE1/AE3, DAKO)*	No staining
EMA *(clone E29, DAKO)*	Rather diffuse staining of spindle-shaped cells
Smooth-Muscle Actin *(clone 1A4, CELL MARQUE)*	Staining of rare cells, staining of vascular structures
Caldesmone *(clone h-CD, DAKO)*	No staining
Desmine *(clone D33, DAKO)*	No staining
CD34 *(clone QBend-10, DAKO)*	No staining of spindle-shaped cells, staining of vascular structures
ERG *(clone EPR3864, VENTANA)*	No staining of spindle-shaped cells, staining of vascular structures

Overall, the morphological features and immunohistochemical profile supported meningothelial differentiation and were in favor of a WHO grade 1 meningioma, within the limitations of the biopsy specimen. Although S100 protein was expressed, the absence of SOX10 expression did not support a conventional Schwannian peripheral nerve sheath tumor. CD34 and ERG were negative in the spindle tumor cells, highlighting only background vascular structures. This finding argued against vascular differentiation and did not support other CD34-positive spindle-cell neoplasms. The lack of desmin and caldesmon expression argued against smooth muscle differentiation. Taken together, the morphologic and immunohistochemical findings supported the diagnosis of a World Health Organization (WHO) grade 1 meningioma according to the 2021 WHO classification, acknowledging the inherent limitations of a biopsy specimen.

**Fig. 2:**
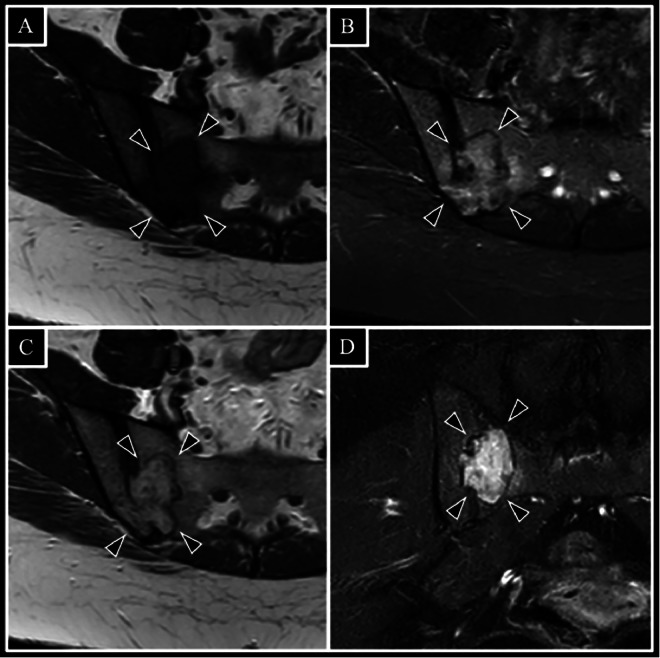
MRI images showing a right-sided sacroiliac mass. (A) T1-weighted sequence; (B) STIR-weighted sequence; (C) T1-weighted postgadolinium sequence; (D) T1 FS postgadolinium sequence, coronal reconstruction; black arrowheads exhibit the meningioma.

Radiologic surveillance with CT at 1-year follow-up was instituted. CT modality was chosen to manage size, cortical erosion and for its better accessibility than MRI in our center. The control examination demonstrated strict morphologic and dimensional stability of the lesion, supporting the benign nature of this WHO grade 1 meningioma (Fig. 2). In the absence of symptoms and given the radiologic stability, we pursued conservative management. The subsequent development of symptoms and/or the appearance of imaging features suggestive of aggressive behavior may prompt reconsideration of the indication for surgery.

## Discussion

Spinal meningiomas are well-defined tumors that predominantly arise in the thoracic intradural compartment. In contrast, sacral involvement—particularly in a purely extradural form—is exceptional. Their rarity, combined with frequently subtle or absent symptoms and nonspecific imaging characteristics, makes preoperative identification particularly challenging.

The pathophysiology of extradural meningiomas remains incompletely understood [11,12]. The most widely accepted hypothesis involves proliferation of ectopic arachnoid cell rests located along nerve root sheaths beyond the dural sac. In the sacral region, the complex foraminal anatomy and the relatively long course of the sacral nerve roots may favor extradural growth with secondary osseous involvement. In our case, the mass is located close to the dorsal ramus of S1. Other proposed mechanisms include embryologic displacement of arachnoid cells or meningothelial differentiation from mesenchymal progenitor cells; however, these theories have not been definitively demonstrated [13].

In the present case, the absence of symptoms, the histologic and immunophenotypic findings, the low proliferative index, and the radiologic stability at 1-year follow-up are all consistent with the diagnosis of a WHO grade 1 meningioma.

This case demonstrates an exceptional biopsy-confirmed meningioma in an extradural sacroiliac mass. Intracranial meningiomas often demonstrate imaging features that are highly suggestive—if not diagnostic—despite their potential radiologic heterogeneity. In contrast, in our case, the presentation was entirely nonspecific, both in terms of imaging characteristics and topography. This observation underscores the importance of considering meningioma in the differential diagnosis of sacral and sacroiliac masses identified on CT and MRI, including in asymptomatic patients.

## Patient consent

The authors declare that informed consent was obtained from the patient for the publication of this case report and any accompanying images.
